# P-1948. Differential Patterns of Protection from Natural Infection against SARS-CoV-2 Reinfection in the Pre-Omicron and Omicron Eras

**DOI:** 10.1093/ofid/ofae631.2107

**Published:** 2025-01-29

**Authors:** Hiam Chemaitelly, Houssein H Ayoub, Laith J Abu-Raddad

**Affiliations:** Weill Cornell Medicine - Qatar, Doha, Ad Dawhah, Qatar; Weill Cornell Medicine - Qatar, Doha, Ad Dawhah, Qatar; Weill Cornell Medicine, Doha, Ad Dawhah, Qatar

## Abstract

**Background:**

SARS-CoV-2 infection protection against reinfection can be compromised by emergence of immune-evasive variants.

**Methods:**

A national, matched, test-negative, case-control study was conducted on Qatar's population between February 5, 2020-February 12, 2024, to assess the level and durability of protection offered by omicron infection against omicron reinfection, compared to the protection provided by pre-omicron infection against pre-omicron reinfection. Subgroup and sensitivity analyses were performed.

**Results:**

Effectiveness of pre-omicron infection against pre-omicron reinfection was 81.1% (95% CI: 80.4-81.8%). Effectiveness was 81.3% (95% CI: 80.6-82.1%) in the first year after the previous infection and 79.5% (95% CI: 77.1-81.5%) thereafter. In contrast, effectiveness of omicron infection against omicron reinfection was only 53.6% (95% CI: 52.1-55.0%) and demonstrated rapid waning. Effectiveness declined from 81.3% (95% CI: 79.6-82.9%) within the first 3-6 months post-infection to 59.8% (95% CI: 57.8-61.7%) in the subsequent 3 months, further dropping to 27.5% (95% CI: 22.7-32.0%) within the next 3 months, and reaching only 4.8% (95% CI: -2.7-11.8%) after one year. Effectiveness against symptomatic reinfection and for both unvaccinated and vaccinated individuals mirrored these patterns. Effectiveness of infection in preventing severe, critical, or fatal COVID-19 upon reinfection remained nearly 100% in both pre-omicron and omicron eras, showing no signs of waning over time.

**Conclusion:**

SARS-CoV-2 natural immunity shows a dynamic interplay between host immunity and viral evolution, resulting in contrasting reinfection patterns before and after omicron emergence. These patterns suggest a shift in evolutionary pressures, with intrinsic transmissibility driving adaptation pre-omicron and immune escape becoming dominant post-omicron.

**Disclosures:**

All Authors: No reported disclosures

